# An Osteoconductive, Osteoinductive, and Osteogenic Tissue-Engineered Product for Trauma and Orthopaedic Surgery: How Far Are We?

**DOI:** 10.1155/2012/236231

**Published:** 2011-10-25

**Authors:** Wasim S. Khan, Faizal Rayan, Baljinder S. Dhinsa, David Marsh

**Affiliations:** University College London Institute of Orthopaedics and Musculoskeletal Sciences, Royal National Orthopaedic Hospital, Stanmore, Middlesex, London HA7 4LP, UK

## Abstract

The management of large bone defects due to trauma, degenerative disease, congenital deformities, and tumor resection remains a complex issue for the orthopaedic reconstructive surgeons. The requirement is for an ideal bone replacement which is osteoconductive, osteoinductive, and osteogenic. Autologous bone grafts are still considered the gold standard for reconstruction of bone defects, but donor site morbidity and size limitations are major concern. The use of bioartificial bone tissues may help to overcome these problems. The reconstruction of large volume defects remains a challenge despite the success of reconstruction of small-to-moderate-sized bone defects using engineered bone tissues. The aim of this paper is to understand the principles of tissue engineering of bone and its clinical applications in reconstructive surgery.

## 1. Introduction

Bone is a highly vascularised tissue that constantly undergoes remodelling as a result of the balance between the activities of the osteoclasts and the osteoblasts, which allows adaptation to mechanical stresses, maintenance of bone health, and repair of small injuries. A recent study demonstrated that the coupling between osteoclastic bone resorption and osteoblastic bone formation is needed for bone homeostasis [1]. Because of the potential of bone to spontaneously regenerate, most small bone lesions, such as fractures, heal well with conventional therapy or surgery. During bone repair, the osteogenic process, under the influence of bone-derived bioactive factors, commences after the inflammatory phase and is initiated by precursor cells from the periosteum adjacent to the fracture site. This generates hard callus by intramembranous bone formation. An autologous bone graft or bone substitute is often required to assist in the healing of an extensive traumatic or postsurgical bone defect and of osseous congenital deformities. The majority of bone formation, however, is by enchondral ossification of the soft callus that appears after infiltrated mesenchymal cells are induced to chondrogenesis. This improved understanding of repair, and regeneration has helped with the development of orthopaedic tissue engineering [2].

Historically, a variety of substitutes like celluloid, aluminium, gold, vitallium, tantalum, stainless steel, titanium, methyl methacrylate resins, polyethylene, silicone elastomers, and hydroxyapatite ceramics have been tried [3]. The main concerns with the use of these synthetic materials for bone reconstruction were their inability to vascularise, integrate, and undergo remodelling. This may result in structural failure of the implant under load or pathological changes in the surrounding bone, as seen in stress shielding [4]. The other issues are inflammatory scarring, neoproliferative reaction in the adjacent tissues and infection [5]. Because of their high osteoinductive potential and remodelling characteristics, bioactive substitutes such as demineralized bone matrix (allogeneic or xenogeneic) have shown promise, despite risk of disease transmission, as well as cost and availability [6]. This led to the evolution of tissue engineering techniques (biologically enhanced allografts, cell-based therapies, and gene-based therapies) to treat bone defects. 

Tissue engineering has been defined as the application of scientific principles to the design, construction, modification, and growth of living tissue using biomaterials, cells, and factors alone and in combination [7]. It involves the use of osteoconductive biomaterial scaffolds, with osteogenic cell populations and osteoinductive bioactive factors. The three components for tissue regeneration are (1) a degradable support or scaffold material; (2) bioactive factors, such as growth factors; (3) cells. The potential for bone tissue engineering therapies in clinical applications is exemplified by the clinical success of recombinant human bone morphogenetic protein-2 for the treatment of fractures [8].

The most promising primary tissue engineering strategies are (1) isolation of mesenchymal stems cells (MSCs), their ex vivo expansion, and seeding onto a scaffold to produce extracellular matrix (ECM) on the scaffold; (2) implantation of an acellular scaffold into the osseous defect [7]. Translation of this technology into practice requires an additional surgical procedure and the time lag for the bone graft to develop in vitro. A variety of novel ex vivo culture techniques have been designed to speed up the cellular production of ECM. Three principal ex vivo culture techniques utilized in bone tissue engineering are growth factor delivery, bioreactor systems, and gene therapy.

## 2. Stem Cells

A stem cell is a cell from the embryo, fetus, or adult that, under certain conditions, can reproduce for long periods. It can also give rise to specialized cells of body tissues and organs. The use of stem cells from the embryo or fetus has many ethical considerations, whereas the use of adult stem cells is generally well accepted by society. An adult stem cell is an undifferentiated or unspecialized cell present in differentiated tissue, which renews itself and becomes specialized to yield all of the cell types of the tissue from which it originated. Their progeny includes both new stem cells and committed progenitors with a more restricted differentiation potential. These progenitor cells in turn give rise to more differentiated cell types. The advantages of using stem cells rather than differentiated cells are a higher proliferative capacity, a higher regenerative potential over time, and the ability to allow revascularization of the avascular scaffold. Cells with osteoprogenitor features have been isolated from several tissues including periosteum, bone marrow, adipose tissue, and retina. The choice of source depends on accessibility, frequency of cells, and information of a particular cell system.

The sources of osteogenic human cells are primary cells, MSCs, embryonic stem cells, and induced pluripotent stem cells. We use cells after considering various factors like proliferation potential, osteogenicity, vasculogenicity, the homogeneity, and the phenotype stability, as well as cell safety after implantation. Differentiation of these cells can be obtained in vitro by changing the culture conditions after their expansion or by providing a new physiological microenvironment in the transplant area in vivo. The process involves the isolation of cells, with expansion in vitro culture and enrichment of appropriate cell type for enhanced bone formation, integration of the cells with host tissues, and expression of stable osteogenic phenotype. 

Primary osteogenic cells can be derived from adult bone tissue and periosteum [9–14]. Due to donor site morbidity [15–17] and limited proliferation of primary cells, preparation of large autologous grafts from primary bone or periosteum-derived cells would be difficult [18–20]. Research suggests that stem cells derived from bone marrow (BMSC) can be expanded for a significant number of cell doublings without cell senescence. The bone marrow is a reservoir of multipotent stem cells for mesenchymal tissues that can differentiate into fibroblastic, osteogenic, adipogenic, and reticular cells [21]. Expansion of stem cells using bone marrow aspirates depends on the donor age, volume, and technique. Although, it has been demonstrated that BMSCs can be culture expanded to large numbers [21], the osteogenic potential of BMSCs is maintained in older individuals [22], and appropriate conditions in vitro (e.g., culture on collagen substrate and growth factor supplementation of culture media) [23, 24] can help maintain cell differentiation potential [25, 26].

Adipose tissue stem cells (ASCs), due to their accessibility and potential for differentiation into osteogenic cells, represent another attractive source for bone tissue engineering. The number of cells produced by expansion is influenced by the tissue harvesting procedure, as well as the site of tissue harvesting, for example, arm, thigh, abdomen, and breast [27, 28]. ASCs undergo similar mesenchymal lineage specific differentiation as BMSC. They also display similar surface antigen. The principle advantages of ASCs use are that they exist in abundant numbers, can be obtained with minimal donor morbidity, their proliferative capacity is unaffected by age, and they have the ability to regenerate bone in critical sized bone defects [29, 30].

Takahashi and Yamanaka [31] described the use of induced pluripotent stem (IPS) cells. The main concern in their conceptualisation was the risk of viral integration into the recipient genome. However, this was allayed by Okita et al. [32] producing virus-free IPS cells from embryonic fibroblasts. Another concern is the time frame taken for extended ex vivo culture to produce sufficient number of IPS cells from fibroblasts. Due to the availability in larger numbers, ASCs-derived IPS cells have the potential to address this issue. 

Human embryonic stem cells (ESCs), isolated by Thomson et al. [33], have unlimited potential for proliferation in vitro and they can form any tissue in the body [34]. ESCs are commonly derived from the inner cell mass (ICM) of preimplantation stage blastocysts [34]. They can be either feeder dependent or independent. Due to the exposure of ESCs to animal components, they pose a serious risk of transmitting serious pathogens, and thus extensive screening is warranted prior to therapeutic applications. 

Bone formation is further controlled by engineering adult stem cells to express genes like bone morphogenetic proteins (BMP2, BMP4, and BMP7), core binding factor *α*1 (Cbfa1), vascular endothelial growth factor (VEGF), and noggin [35]. In addition, human bone marrow osteoprogenitor cells can be isolated and enriched using monoclonal antibodies as selective markers, such as STRO-1, from a CD34+ fraction, SB-10 (reacting with ALCAM), SH-2 (reacting with CD105), and HOP-26 (reacting with CD63) [12, 13]. Fibroblast growth factor-2 (FGF-2) supplementation to the culture medium promotes cell proliferation and maintains their multilineage potential during expansion [14]. These cells can be combined with a suitable scaffold and used as an alternative to conventional bone autograft. The transplanted osteogenic stem cells can immediately begin to proliferate and lay down new bone matrix without removing the old matrix present in the autograft. The development of these cell-based technologies may result in decreased use of dead bone from conventional bone banks to induce new bone formation.

## 3. Scaffold

A key component in tissue engineering for bone regeneration is the scaffold that serves as a template for cell interactions and the formation of bone extra cellular matrix to provide structural support to the newly formed tissue [6, 7]. Mesenchymal stem cells alone are unlikely to be sufficient for bone regeneration. Although marrow injections are simple and provide a reduced risk of morbidity, for large skeletal defects, a scaffold of appropriate shape, size, and mechanical competence is required for bone reconstruction [2]. The use of the scaffold or matrix is not only in controlling growth factor and cell delivery but also to provide a structural template to fill the tissue lesion [22]. Ideally, the scaffold should facilitate cell infiltration, matrix deposition, and cell attachment and consist of osteoconductive materials such as bone protein and hydroxyapatite. They should be able to allow load bearing and stimulate osteogenesis. The scaffolds could be naturally occurring, synthetic polymers, or bioceramics. Biodegradable scaffolds provide the initial structure and stability for tissue formation but degrade as tissue forms, providing background for matrix deposition and tissue growth [15–18]. They can be used alone or in combination with growth factors or osteoconductive materials [7].

The scaffold aims to mimic the extracellular matrix in a regenerating bone environment. It has to be informative to the cells as well as provide mechanical support [7]. A biomaterial should easily integrate with the adjacent bone and favour new tissue ingrowth (osteoconduction). It should allow colonization by the host blood vessels, be biocompatible and resorbable.

Various synthetic biomaterials like inorganic ceramics (e.g., hydroxyapatite, coralline-derived hydroxyapatite, tricalcium phosphate, calcium sulphates, glass ceramics, calcium phosphate-based cements, and bioglass), metals, and synthetic biodegradable polymer composites have been investigated for their potential as bone scaffold materials. Calcium-phosphate ceramics were introduced more than 40 years ago as bone substitutes. The most common types of calcium-phosphate materials investigated for synthetic bone scaffold development are hydroxyapatite (HA), tricalcium phosphate (TCP), biphasic calcium phosphates (BCP), and bioglasses. From a functional perspective, you can divide these into rapidly resorbing, slowly resorbing, and injectable ceramics. TCP is a classic example of rapidly resorbing ceramic; it has got greater solubility than HA. Due to their porosity, TCP granules are a better option than the bulk form [35], while HA resorbs slowly, which is clinically a disadvantage. Composite modification of HA matrices has been tried to increase the resorption, for example, composite of HA and calcium carbonate and BCP. Injectable calcium phosphate cements were also in vogue. They are mainly composed of *α*-TCP, dibasic dicalcium phosphate and tetra calcium phosphate. Clinically, they have been used in treatment of distal radius fractures [35–37]. The main disadvantage of their clinical relevance as synthetic bone scaffolds is due to their inherent brittleness [38]. 

However, due to their physiochemical properties, biocompatibility, and controllable biodegradability, polymers have emerged as the principal material in bone tissue engineering. The most frequently investigated polymers are polylactic acid (PLA) and polyglycolic acid (PGA) [39–41]. Numerous polymers were used as scaffold materials in the past decade for bone regeneration like poly(a-hydroxy esters), poly(ethylene glycol), polydioxanone, poly(orthoesters), polyanhydrides, polyurethanes, and poly(propylene fumarate) [42]. To gain more control over the degradation rate, hydrophobicity, crystallinity, and biological functionality, researchers designed composite polymers in a chemical process called copolymerization where multiple constituents are combined resulting in a new material with desirable properties from each constituent [43]. Undoubtedly, the most commonly utilized copolymer for bioactive molecule encapsulation and release for bone tissue engineering is the copolymer poly(lactic acid-co-glycolic acid) (PLGA) [43]. The inherent deficiency of the compressive modulus in polymers may be reduced through integration of high modulus micro- and/or nanoscale constituents within the polymer matrix [43]. The most commonly researched constituent in polymer composites for bone scaffolds is micro- or nanoscale HA particles [44, 45]. Tensile strength, modulus, and crack resistance of polymers are improved by dispersing high modulus micro- or nanoscale constituents [15–17, 46]. Whereas drawbacks for utilizing natural polymers like collagen, glycosaminoglycan, fibrin, and silk include infection, fixed degradation rates, and immunogenicity. Gel-like matrices such as fibrin have been used for cell immobilization in combination with other scaffolds [47]. Currently, computer-assisted design/computer-assisted manufacturing (CAD/CAM) and rapid prototyping techniques allow the generation of custom-made scaffolds for cell delivery that fit into certain bone defects [22, 48].

There is a large number of osteogenic proteins that stimulate proliferation and differentiation of osteogenic cells in vitro and in vivo. Some osteogenic factors have been cloned and are commercially available as recombinant proteins. The most potent osteoinductive factors are bone morphogenetic protein (BMP). BMPs belong to the TGF-*β* family [49]. BMP-2 and BMP-7 are being clinically applied for fractures and nonunions [8, 50]. They have a short half-life, so local BMP delivery systems either require a high concentration bolus dose or sustained delivery for bone tissue engineering [51]. However, high BMP concentrations are associated with increased osteoclastic activity and bone resorption [23, 24]. Other options are the direct implantation of a carrier that allows slow release or gene-based therapies, where a transgene for BMP expression is delivered to progenitor cells [52, 53]. Collagen carriers have historically been and remain the primary delivery system for BMPs to clinical defects. Because collagen has got poor BMP retention, higher BMP drug concentrations are required. Another concern is the potential for an immunogenic response or disease transfer from animal-derived collagen (e.g., variants of Creutzfeldt-Jacob disease or other prion-related diseases) [54]. A number of synthetic biomaterials have been proposed for BMP, such as inorganic ceramics, metals, and synthetic biodegradable polymers. Many of these materials are poorly biodegradable and radiopaque, whereas synthetic biodegradable polymers are mouldable and radiolucent. These characteristics make it easier to assess radiographic growth [55, 56].

Tissue engineering strategies aim at controlling the behaviour of individual cells to stimulate tissue formation. Currently, tissue-engineered bone is constructed using a perfusion bioreactor in vitro. Several different bioreactors have been investigated for tissue-engineering applications. Among these bioreactors are the spinner flask rotating wall vessel reactors and the flow perfusion culture bioreactors. Flow perfusion culture offers several advantages, notably the ability to mitigate both external and internal diffusional limitations as well as to apply mechanical stress to the cultured cells. In the perfusion culture, fluid flow can exert shear stress on the cells seeded on scaffold, improving the mass transport of the cells. Bioreactor systems of a variety of designs have also been utilized to enhance the in vitro performance of osteogenic cells before implantation. Bioreactors simulate the 3D dynamic and mechanical in vivo environment and are designed to provide cells seeded deep within a scaffold with all necessary nutrients and biological cues to survive, proliferate, differentiate, and produce ECM [57, 58]. Sikavitsas et al. [59] demonstrated proof of this concept by showing that after 16 days of culture, MSC-produced ECM was uniformly distributed in 3D scaffolds cultured in a flow profusion bioreactor, whereas the ECM was limited to the periphery in the case of standard static culture condition. Janssen et al. [60] demonstrated that direct perfusion bioreactor system is capable of producing clinically relevant volumes of tissue-engineered bone in a bioreactor system, which can be monitored on line during cultivation. 

In summary, many factors can influence the osteoblastic differentiation of marrow stromal cells when cultivated on three-dimensional tissue engineering scaffolds. In creating ideal bone tissue engineering constructs consisting of a combination of a scaffold, cells, and bioactive factors, a flow perfusion bioreactor is a much more suitable culture environment than static culture in well plates. The bioreactor eliminates mass transport limitations to the scaffold interior and provides mechanical stimulation to the seeded cells through fluid shear [61]. Scaffold properties such as pore size impact cell differentiation, especially in flow perfusion culture. In addition, the bone-like ECM created by the in vitro culture of marrow stromal cells on porous scaffolds creates an osteoinductive environment for the differentiation of other marrow stromal cell populations. Therefore, bone tissue engineering constructs created by in vitro culture have excellent potential for bone regeneration applications in the clinical setting.

## 4. Clinical Outcomes

In literature, there are numerous studies demonstrating the effectiveness of bone tissue engineering techniques in the rodent model; however, little has been produced demonstrating its role in reconstructing osseous defects in larger animals. Petite et al. [62] investigated the role of in vitro expanded MSCs on a coral scaffold in large segmental bone defects in sheep. The study compared this technique with using the scaffold alone and the use of scaffolds with fresh bone marrow. With the tissue-engineered technique, clinical union was demonstrated in three out of seven bone defects, compared with no evidence of clinical union in any of the defects that were left empty or filled with scaffold alone. 

With regards to the use of tissue engineering strategies in human bone reconstruction, published literature is sparse. Schimming and Schmelzeisen [63] reported the use of periosteum-derived tissue-engineered bone for the augmentation of the posterior maxilla. At three-month followup, eighteen out of twenty-seven patients demonstrated an excellent clinical, radiological, and histological outcome. Marcacci et al. [64] reported the use of ex vivo expanded bone marrow-derived MSCs implanted on a macroporous hydroxyapatite scaffold in four patients with large bone defects. One patient had a four-centimetre bone defect of the mid-diaphysis of the tibia following unsuccessful bone lengthening, another had traumatic loss of four centimetres of bone from the distal diaphysis of the ulna, the third patient had a seven centimetres bone defect of the humerus following a fracture and the final patient had six centimetres of traumatic bone loss from the ulna. The scaffolds were of the shape and size to fit each defect when implanted. External fixation was used for mechanical stability and removed after 6.5 months for the first patient, at 6 months for the second patient, at 13 months in the fourth patient, and 7 months for the final patient. Abundant callus formation along the implants and good integration with the host bones were evident on radiography and computed tomography after 1-2 months. At a minimum of 1-year followup, good integration of the implant to host bone was evident. All the patients reported favourable limb function outcome [64].

## 5. Conclusion

The use of tissue engineering for the reconstruction of bone defects has exciting potential; however, there is much work to be done before this strategy can be considered a serious clinical option [65–71]. The majority of research in MSC-based bone reconstruction has looked at isolation and expansion in vitro of MSCs, their delivery to defect sites and techniques to improve proliferation potential, and direct the MSCs towards osteogenesis using the appropriate factors [72–86].

Whilst animal studies have proven to show some success, the use of tissue engineering to repair bone defects in humans remains a challenge with limited clinical data. The reason for the perceived failure of these strategies in humans is thought to lie with an inadequate vascular supply, leading to cell death of implanted cells. There have also been concerns raised by the poor resorbability of the scaffolds and instability of the scaffold fixation. Whilst much work has been done on the factors involved in tissue engineering, more study is required to improve the key factor of cell survival in human models, such as improving nutrient and oxygen supply. Eventually, randomised controlled trials will be required to determine the effectiveness of tissue engineering approaches to bone reconstruction in humans before clinical use can be considered a viable option.
